# Objectively Measured Physical Activity and Sedentary Time during Childhood, Adolescence and Young Adulthood: A Cohort Study

**DOI:** 10.1371/journal.pone.0060871

**Published:** 2013-04-23

**Authors:** Francisco B. Ortega, Kenn Konstabel, Elena Pasquali, Jonatan R. Ruiz, Anita Hurtig-Wennlöf, Jarek Mäestu, Marie Löf, Jaanus Harro, Rino Bellocco, Idoia Labayen, Toomas Veidebaum, Michael Sjöström

**Affiliations:** 1 “PROmoting FITness and Health through physical activity” research group, Department of Physical Education and Sports, School of Sport Sciences, University of Granada, Granada, Spain; 2 Department of Biosciences and Nutrition at NOVUM, Karolinska Institutet, Huddinge, Stockholm, Sweden; 3 Department of Chronic Diseases, Centre of Behavioural and Health Sciences, National Institute for Health Development, Tallinn, Estonia; 4 Department of Psychology, Estonian Centre of Behavioural and Health Sciences, University of Tartu, Tartu, Estonia; 5 Division of Epidemiology and Biostatistics, European Institute of Oncology, Milan, Italy; 6 School of Health and Medical Sciences/Clinical Medicine, Örebro University, Örebro, Sweden; 7 Department of Coaching Sciences, Faculty of Exercise and Sport Sciences, Centre of Behavioral and Health Sciences, University of Tartu, Tartu, Estonia; 8 Department of Medical Epidemiology and Biostatistics, Karolinska Institutet, Stockholm, Sweden; 9 Department of Statistics and Quantitative Methods, University of Milano-Bicocca, Milan, Italy; 10 Department of Nutrition and Food Science, University of the Basque Country, Universidad del País Vasco/European Humanities University, Vitoria, Spain; 11 National Institute for Health Development, Centre of Behavioural and Health Sciences, Tallinn, Estonia; University of Sao Paulo, Brazil

## Abstract

**Background:**

To know how moderate-to-vigorous physical activity (MVPA) and sedentary time change across lifespan periods is needed for designing successful lifestyle interventions. We aimed to study changes in objectively measured (accelerometry) MVPA and sedentary time from childhood to adolescence and from adolescence to young adulthood.

**Methods:**

Estonian and Swedish participants from the European Youth Heart Study aged 9 and 15 years at baseline (N = 2312) were asked to participate in a second examination 6 (Sweden) to 9/10 (Estonia) years later. 1800 participants with valid accelerometer data were analyzed.

**Results:**

MVPA decreased from childhood to adolescence (−1 to −2.5 min/d per year of follow-up, P = 0.01 and <0.001, for girls and boys respectively) and also from adolescence to young adulthood (−0.8 to −2.2 min/d per year, P = 0.02 and <0.001 for girls and boys, respectively). Sedentary time increased from childhood to adolescence (+15 and +20 min/d per year, for girls and boys respectively, P<0.001), with no substantial change from adolescence to young adulthood. Changes in both MVPA and sedentary time were greater in Swedish than in Estonian participants and in boys than in girls. The magnitude of the change observed in sedentary time was 3–6 time larger than the change observed in MVPA.

**Conclusions:**

The decline in MVPA (overall change = 30 min/d) and increase sedentary time (overall change = 2∶45 h/d) observed from childhood to adolescence are of concern and might increase the risk of developing obesity and other chronic diseases later in life. These findings substantially contribute to understand how key health-related behaviors (physical activity and sedentary) change across important periods of life.

## Introduction

An expert committee on behalf of the American Heart Association and the American College of Sport Medicine has reported the multiple health benefits of an active lifestyle[1]. Physical activity (PA) and sedentary behaviors independently predict chronic disease risk and mortality, and they should be considered as separate constructs[2], [3], [4], [5], [6], [7], [8]. In order to design successful interventions and policies to promote PA and reduce sedentary time, there is a need to better understand how PA and sedentary time change over important periods of life. The lack of knowledge in this area is mainly due to the complexity to accurately measure PA and sedentary time. Most of previous research is based on self-reported methods (e.g. questionnaires). However, important drawbacks have been recognized, particularly in pediatric population[9], [10], [11], [12]. Due to the limitations of self-report methods, accelerometry has become the method of choice for objectively measuring PA in free-living children and adolescents[13], [14].

Although population-based cross-sectional studies have reported interesting information about PA and sedentary levels in different age groups[15], [16], [17], [18], [19], [20], [21], [22], [23], [24], only longitudinal studies using objective methods are able to accurately describe changes in PA and sedentary time across lifespan periods. Some studies have recently reported changes in PA and/or sedentary time using accelerometry over relatively short periods in life (2–3 years of follow-up)[25], [26], [27], [28], [29], [30]. The most comprehensive longitudinal study using objective methods reported a decline in PA in US children from 9 to 15 years of age[31], and increase in sedentary time during that period[30]. A recent study reported an increase in objectively measured sedentary time in British children from 12 to 16 years of age[32]. Adolescence is a period involving dramatic physiological and psychological changes; however, the transition to young adulthood implies for many people to leave their family homes (due to university/work), and important lifestyle changes might also occur. Information on changes in PA/sedentary time from adolescence to young adulthood is needed.

The European Youth Heart Study (EYHS) was one of the first epidemiological studies using accelerometry at population level to objectively measure PA and sedentary time in children and adolescents in 1998. Estonian and Swedish participants from the original EYHS participated in a second examination 6 to10 years later. These data provide a unique opportunity to study changes in PA and sedentary time from childhood to adolescence and from adolescence to young adulthood. This contributes to the previous literature by addressing the following scientific questions: 1) Is the change in PA and/or sedentary time from childhood to adolescence more pronounced than from adolescence to young adulthood? 2) Are there gender differences in the pattern of change in PA and/or sedentary time during the study periods? 3) Is the magnitude of the change in PA different from that observed in sedentary time? 4) Are the changes in PA and/or sedentary time similar in weekdays compared with weekend days?

## Materials and Methods

### Study sample and design

**Figure 1:**
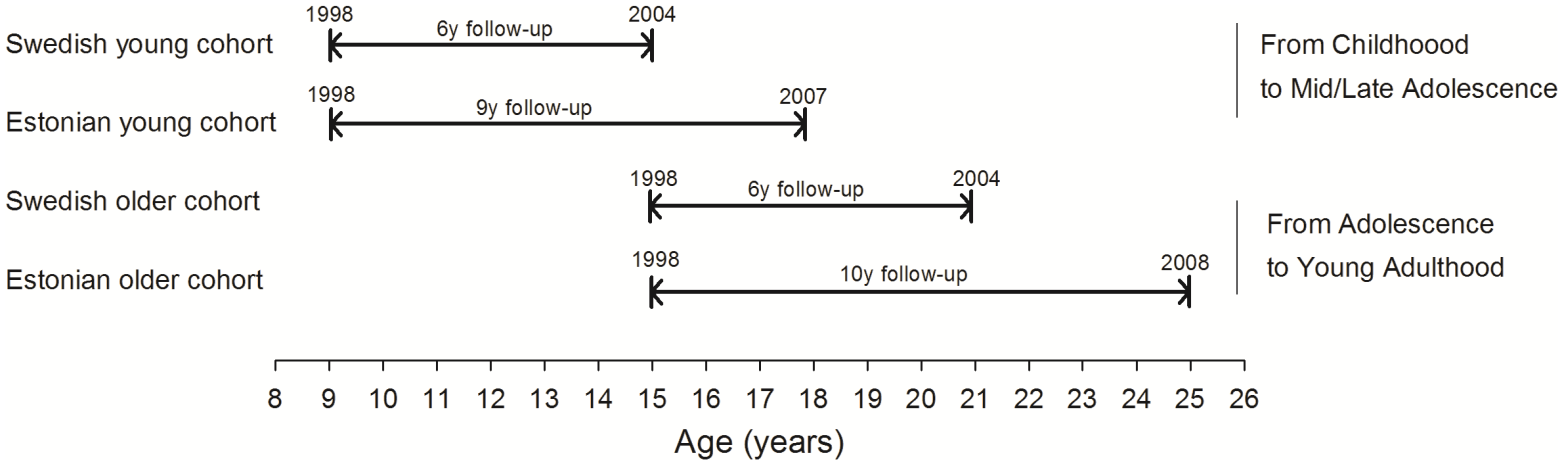
Study design.

This study included Estonian and Swedish children and adolescents who participated in the EYHS in 1998–1999 (Baseline). In Estonia, the city of Tartu and its surrounding rural area was the geographical sampling area. In Sweden, seven municipalities in the Stockholm area and one in Örebro were chosen for data collection. Study design, selection criteria and sample calculations have been reported elsewhere[33], [34]. All the participants were invited to a second examination 6 and 9–10 years later, for Sweden and Estonia respectively. The study design is depicted in **Figure 1**. Differences in follow-up period between countries are due to practical and funding reasons. The follow-up assessment in the Estonian cohort was carried out as part of the longitudinal Estonian Children Personality Behaviour and Health Study[35].

A total of 1176 Estonian children (9 y) and adolescents (15 y) and a total of 1136 Swedish children (9 y) and adolescents (15 y) participated in the baseline examination (53.8% girls). Participants were mostly white (Caucasian), i.e. 100% and 88% in Estonia and Sweden respectively. Participants with valid accelerometer data at least at one time point (i.e. baseline, follow-up or both) were included in the study, as per previous longitudinal studies[27], [31]. A total of 1800 participants (77.9%), 811 boys and 989 girls, met this criterion. Percentage of participants included in the analyses was 78.9% in Estonia and 76.8% in Sweden. Girls were 23% more likely to be included in the study compared with boys (P = 0.04). Likewise, participants with highly educated mothers (university level) were 29% more likely be included in the study (P = 0.03). Included participants were younger than excluded participants (mean age = 12.3 *vs.* 13.4 years, respectively; P<0.001). After adjustment for age and sex, no difference between participants included *vs.* excluded was observed in weight, height or body mass index (BMI) (All P≥0.1). Number of participants by country, age cohort and sex at both time points are shown in **Table 1**.

**Table:** **Table 1.** Characteristics of the participants.

	Swedish cohort										Estonian cohort							
	Young cohort					Older cohort					Young cohort				Older cohort			
Baseline data*	Boys		Girls			Boys		Girls			Boys		Girls		Boys		Girls	
**Weight (kg)**	33.5	(6.3)	33.7		(6.7)	63.9	(11.1)	57.6		(9.1)	31.7	(5.5)	31.1	(6.3)	61.5	(10.6)	54.9	(7.9)
**Height (cm)**	139.3	(6.0)	139.0		(6.7)	175.3	(7.5)	165.1		(6.5)	137.6	(6.4)	137.1	(6.7)	173.6	(7.8)	164.7	(6.2)
**Body mass index (kg/m^2^)**	17.2	(2.4)	17.3		(2.4)	20.7	(2.8)	21.1		(2.8)	16.7	(1.9)	16.4	(2.3)	20.3	(2.7)	20.2	(2.5)
**Mother’s education (%)** University level	27.6		33.3			35.7		43.0			30.1		33.7		29.6		30.4	
**Sexual maturation (%)**	99/1/0/0/0		57/40/3/0/0			0/1/4/18/77		0/0/6/48/46/0			86/14/0/0/0		76/21/3/0/0		1/4/19/31/45		0/1/13/39/47	
Stages I/II/III/IV/V																		
Baseline/Follow-up data																		
**N** † Baseline	180		213			157		203			214		220		156		223	
Follow-up	107		133			62		106			114		149		63		90	
**Age (y)**																		
Baseline	9.6	(0.3)	9.5	(0.4)		15.6	(0.4)	15.6	(0.4)		9.6	(0.5)	9.4	(0.5)	15.5	(0.6)	15.3	(0.5)
Follow-up	15.5	(0.7)	15.5	(0.4)		21.6	(0.4)	21.5	(0.7)		18.3	(0.5)	18.3	(0.6)	25.3	(0.5)	25.1	(0.5)
**Weekly MVPA (min/d)**																		
Baseline	99.9	(48.0)	73.3	(30.4)		68.3	(34.1)	58.3	(25.2)		79.8	(55.1)	60.8	(42.8)	66.7	(47.9)	48.1	(35.1)
Follow-up	52.4	(23.1)	44.2	(19.7)		45.6	(29.9)	39.8	(20.7)		59.1	(30.7)	52.3	(29.6)	42.5	(27.4)	35.3	(26.2)
**Weekly Sedentary (min/d)**																		
Baseline	307.9	(104.7)	304.5	(67.7)		461.2	(100.7)	467.6	(80.0)		326.1	(134.3)	359.5	(142.7)	436.2	(136.6)	478.4	(114.7)
Follow-up	485.5	(78.6)	482.0	(67.3)		483.0	(96.6)	468.8	(88.6)		506.4	(95.1)	496.1	(76.9)	455.1	(103.4)	469.0	(91.1)
**Weekday MVPA (min/d)**																		
Baseline	94.0	(46.6)	69.3	(30.4)		56.0	(27.7)	51.5	(22.8)		75.2	(51.7)	57.4	(40.2)	58.9	(44.2)	45.6	(32.7)
Follow-up	45.7	(20.6)	40.1	(16.4)		37.7	(18.4)	33.3	(17.0)		53.8	(28.4)	49.0	(26.9)	37.3	(23.9)	32.5	(25.2)
**Weekday Sedentary (min/d)**																		
Baseline	311.6	(108.1)	310.7	(74.1)		471.8	(107.8)	481.5	(92.3)		341.1	(138.1)	379.7	(147.6)	453.0	(144.2)	505.5	(120.7)
Follow-up	497.8	(85.8)	502.7	(72.0)		495.3	(109.6)	472.7	(92.6)		525.5	(114.1)	520.9	(90.8)	463.4	(115.2)	480.6	(101.5)
**Weekend MVPA (min/d)**																		
Baseline	77.5	(47.6)	55.9	(31.1)		46.3	(39.9)	38.6	(24.4)		68.2	(56.5)	51.8	(42.7)	46.3	(42.6)	33.0	(30.6)
Follow-up	34.0	(26.3)	31.0	(19.9)		30.6	(21.6)	33.6	(22.8)		34.5	(31.0)	32.5	(29.0)	33.4	(27.9)	24.1	(21.8)
**Weekend Sedentary (min/d)**																		
Baseline	298.7	(116.8)	288.9	(88.8)		434.6	(122.4)	432.6	(96.3)		288.6	(144.1)	309.1	(145.3)	394.3	(140.7)	410.6	(123.2)
Follow-up	454.8	(97.2)	430.4	(89.0)		452.4	(98.9)	459.1	(107.5		458.5	(113.6)	434.0	(95.8)	434.3	(126.1)	440.0	(105.6)

Data are means (standard deviation), unless otherwise indicated. MVPA indicates moderate-to-vigorous PA. Weekly indicates the full week average; weekday, Monday to Friday; and weekend Saturday and Sunday. *These baseline data were available for all the participants included in the analyses (N = 1800), with 4 missing data for anthropometrics and 108 missing values for maternal education. † The same sample size was available for all the activity/sedentary variables.

### Ethics statement

The study protocol was performed in accordance with the ethical standards laid down in the 1961 Declaration of Helsinki (as revised in 2000), and approved by the Research Ethics Committees of University of Tartu (no. 49/30-199), Örebro County Council (no. 690/98) and Huddinge University Hospital (no. 474/98). Participants and/or one parent/legal guardian provided written informed consent, after procedures were explained.

### Baseline physical examination and maternal education

Height (cm) and weight (kg) were measured by trained staff using standardized procedures[33], [34], and BMI (kg/m^2^) was calculated. Sexual maturation status was estimated by a trained researcher of the same sex as the participant, according to Tanner and Whitehouse (pubertal stages from I to V) [36]. Maternal education at baseline was used as an indicator of socioeconomic status, consistent with previous studies[37], [38], [39]. The highest level of education was reported by the mothers and coded as 0 = below university and 1 = university.

### Physical activity/sedentary time assessment

Physical activity and sedentary time were objectively measured using Actigraph accelerometers (LLC, Pensacola, Florida). Time spent in sedentary time was defined using the standard cut point of ≤100 counts/min[19], [22], [23], [40]. Time spent in activities of moderate or higher intensity (i.e. MVPA) was defined using the cut point of ≥2000 counts/min [6], [19], [41], [42]. A detailed description of the methods used for accelerometer data analysis is provided as **Methods S1**, and the average number of valid days and registered time in each country, age-cohort and sex group, weekdays and weekend days separately, are presented as **Table S1**.

### Statistical analyses

All the analyses were conducted using Stata 12 (StataCorp LP, College Station, TX, USA). Change in MVPA and sedentary time (main outcomes) were analyzed separately using Mixed Effect Models (also called multilevel modeling). An advantage of Mixed modeling is that it allows including subjects with different number of observations, maximizing the power in the analyses. Models were built considering both random intercepts and random slopes, in order to model individual change over time. Exact age at baseline and follow-up was used as a time varying covariate in the models, and age was centered at baseline. All the analyses were adjusted for accelerometer registered time, number of valid days and country. Interaction terms for age*country and age*sex were entered into the models to test whether changes in MVPA and sedentary time significantly differed by country and sex. Since, significant interactions were found with sex for most of the study variables, and levels and patterns of PA and sedentary time are known to differ by sex[19], [31], [43], all the results are presented for boys and girls separately.

The analyses were repeated using sex- and age-specific standardized scores, i.e. z-score =  (individual observation *minus* mean value)/standard deviation. Analyses on standardized scores allow direct comparison between the change observed in MVPA and the change observed in sedentary time. Main analyses were conducted on weekly (weekdays were weighed by 5 and weekend days by 2) MVPA and sedentary time. Further analyses are also presented for weekdays (Monday to Friday) and weekend days (Saturday and Sunday).

## Results

Characteristics of the study sample as well as levels of MVPA and sedentary time at both time points by country, age cohort and sex are shown in **Table 1**.

The time spent in MVPA significantly decreased 2.5 and 1.0 min/d in boys and girls respectively, per follow-up year from childhood to adolescence (**Table 2**). Slightly less pronounced decrease, yet significant, i.e. −2.2 and −0.8 min/d per year was observed from adolescence to young adulthood. A significant interaction with country was observed from childhood to adolescence (not from adolescence to young adulthood), indicating that MVPA decreased ∼4 minutes more in Swedish participants compared with their Estonian peers (reference group). In secondary analysis, we observed that light PA (defined as 101 to 1999 counts/min) also declined from childhood to adolescence in boys and girls, i.e. −17.6 and −14.3 min/d per year, respectively (P<0.001); without changes from adolescence to young adulthood (P>0.7) (data not shown).

**Table 2:** Mixed effect models examining the change in weekly (weekdays and weekend days weighted average) moderate-to-vigorous physical activity (min/d) from childhood to adolescence and from adolescence to young adulthood in boys and girls.

		Boys				Girls		
Young cohort (N = 960)	Coef.	95% CI		P	Coef.	95% CI		P
Intercept at baseline age (min/d)	30.5	−1.0	62.1	0.058	−3.7	−29.9	22.6	0.785
Age (per year) †	−2.5	−3.5	−1.4	<0.001	−1.0	−1.8	−0.2	0.010
Registered time (min/d)	0.1	0.0	0.1	<0.001	0.1	0.0	0.1	<0.001
Valid days (no.)	−1.7	−5.9	2.5	0.430	0.2	−3.6	4.0	0.928
Country (Estonia = 0, Sweden = 1)	19.4	9.4	29.5	<0.001	11.5	4.6	18.3	0.001
Age*country ‡	−4.4	−6.8	−2.1	<0.001	−3.8	−5.8	−1.8	<0.001

Older cohort (N = 840)	Coef.	95% CI		P	Coef.	95% CI		P
Intercept at baseline age (min/d)	5.9	−31.8	43.5	0.760	−27.2	−53.1	−1.4	0.039
Age (per year) †	−2.2	−3.1	−1.2	<0.001	−0.8	−1.5	−0.1	0.018
Registered time (min/d)	0.1	0.1	0.2	<0.001	0.1	0.1	0.1	<0.001
Valid days (no.)	−8.7	−14.2	−3.3	0.002	−3.1	−7.0	0.9	0.128
Country (Estonia = 0, Sweden = 1)	−1.2	−10.2	7.7	0.788	7.7	2.1	13.3	0.007
Age*country ‡	2.6	−0.2	5.4	0.072	0.0	−2.0	2.0	0.989

† Age was centered on age at baseline. The coefficient (confidence intervals, CI) is interpreted as change in physical activity (min/d) per year of follow-up. Mean (min-max) follow-up period was 7.5 (4.9–9.4) years and 7.9 (5.7–10.3) in the young cohort and older cohort respectively.

‡ The coefficient for age*country interaction term is interpreted as follows: e.g. Coef =  −4.4, physical activity decreased 4.4 min/d more in Swedish participants compared with Estonian participants per year of follow-up.

Sedentary time significantly increased from childhood to adolescence to a yearly rate of 20 and 15 min/d, in boys and girls respectively; being the increase per year in the Swedish participants 8–11 min/d greater than in Estonian participants (**Table 3**). No significant change in sedentary time was observed from adolescence to young adulthood, without differences by country or sex.

**Table 3:** Mixed effect models examining the change in weekly (weekdays and weekend days weighted average) sedentary time (min/d) from childhood to adolescence and from adolescence to young adulthood in boys and girls.

		Boys				Girls		
Young cohort (N = 960)	Coef.	95% CI		P	Coef.	95% CI		P
Intercept at baseline age (min/d)	−155.9	−234.8	−76.9	<0.001	−72.1	−138.8	−5.4	0.034
Age (per year) †	20.0	17.7	22.4	<0.001	15.3	13.4	17.3	<0.001
Registered time (min/d)	0.6	0.5	0.7	<0.001	0.5	0.4	0.6	<0.001
Valid days (no.)	5.7	−5.0	16.5	0.294	7.1	−2.6	16.8	0.151
Country (Estonia = 0, Sweden = 1)	−23.6	−46.5	−0.8	0.043	−59.1	−79.7	−38.6	<0.001
Age*country ‡	7.8	2.0	13.6	0.008	11.3	6.0	16.5	<0.001

Older cohort (N = 840)	Coef.	95% CI		P	Coef.	95% CI		P
Intercept at baseline age (min/d)	68.9	−50.1	187.9	0.257	107.4	15.8	199.1	0.022
Age (per year) †	2.7	−0.2	5.7	0.068	0.8	−1.2	2.9	0.423
Registered time (min/d)	0.4	0.3	0.5	<0.001	0.4	0.3	0.5	<0.001
Valid days (no.)	8.9	−8.2	26.0	0.308	12.5	−2.4	27.5	0.100
Country (Estonia = 0, Sweden = 1)	11.2	−14.9	37.2	0.402	−23.3	−41.7	−4.8	0.014
Age*country ‡	0.1	−8.5	8.6	0.988	−1.4	−8.7	5.9	0.713

† Age was centered on age at baseline. The coefficient (confidence intervals, CI) is interpreted as change in sedentary time (min/d) per year of follow-up. Mean (min-max) follow-up period was 7.5 (4.9–9.4) years and 7.9 (5.7–10.3) in the young cohort and older cohort respectively.

‡ The coefficient for age*country interaction term is interpreted as follows: e.g. Coef = 7.8, sedentary time increased 7.8 min/d more in Swedish participants compared with Estonian participants per year of follow-up.

**Figure 2:**
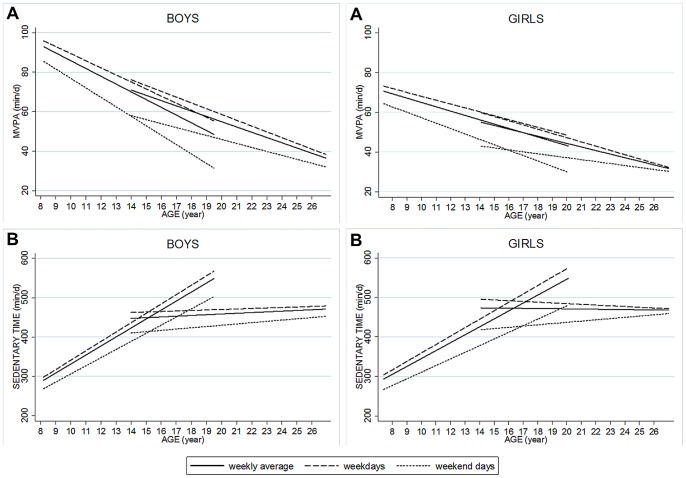
Changes in weekly, weekday and weekend moderate-to-vigorous physical activity (Panel A, MVPA, min/d) and sedentary time (Panel B, min/d). Slopes result from mixed effect models adjusted for age, country, registered time and number of valid days (N = 821 and 989, for boys and girls respectively).

Using the mean follow-up period, as well as country and sex differences on the coefficients observed, we can estimate an overall decrease in MVPA from childhood to adolescence ranging between 8 and 52 min/d (average = 30 min/d), and from adolescence to young adulthood between 6 and 20 min/d (average = 13 min/d). The overall increase from childhood to adolescence in sedentary time ranged between 115 and 209 min/d, i.e. ∼2 h and 3∶30 h/d (average = 2∶45 h). Patterns of change in weekly MVPA and sedentary time, as well as in weekdays and weekends, are graphically represented in **Figure 2**.

Greater changes in weekly MVPA (**Table 2**) and sedentary time (**Table 3**) were observed in boys compared with girls, both from childhood to adolescence and from adolescence to young adulthood (P values for age*sex interactions ranging from 0.01 to <0.001) (data not shown). The results were nearly identical when the models were additionally adjusted for baseline body mass index or maternal educational level (data not shown).

Analyses using weekly MVPA and sedentary time z-scores as outcomes (**Table S2** and **Table S3**), instead of the raw variables, showed that the size of the change in sedentary time was 3–6 times larger than that observed in MVPA from childhood to adolescence (coefficients =  0.12–0.14 *vs* 0.02–0.04, respectively). When the analyses were done separately for weekdays and weekend days (**Table S4** to **Table S7**), the patterns of change observed were similar to those reported for weekly MVPA and sedentary time. Both MVPA and sedentary time was lower in weekend days than in weekdays, and these differences remained over the follow-up period. However, **Figure 2** graphically shows that differences between weekend days and weekdays in levels of MVPA and sedentary time became larger from childhood to adolescence and smaller from adolescence to young adulthood.

## Discussion

Four important findings relevant for health promotion and disease prevention emerged from this prospective study in youth. First, MVPA decreases from childhood to adolescence and from adolescence to young adulthood, yet with slightly lower rate; whereas sedentary time increases only from childhood to adolescence, with no substantial change adolescence to young adulthood. Second, the decline in MVPA and increase in sedentary time is significantly greater in boys than in girls. Third, the magnitude of the change observed in sedentary time was 3–6 times larger than the change observed in MVPA. Fourth, participants spent less time in both MVPA and sedentary time during weekend days compared with weekdays over the whole follow-up period, with the largest differences taking place around adolescence and the smallest at childhood and young adulthood.

It is important to highlight that this study describes changes in two age cohorts. This allows us to draw conclusions on changes from childhood to adolescence and from adolescence to young adulthood, but the results cannot be interpreted as changes from childhood to young adulthood. Nevertheless, until a long follow-up study describes the changes in objectively measured MVPA and sedentary time in a same cohort from 9 years to 25 years, i.e. childhood to young adulthood, the information presented in the current study provides the closest approach to describe how activity/sedentary patterns change over these periods of life.

### Changes in the young cohort *vs.* older cohort

To the best of our knowledge, this is the first time that changes in objectively measured MVPA and sedentary time are described for two different age cohorts followed over a relatively long period (i.e. 6 to 10 years follow-up). This provides a unique opportunity to compare changes in two important periods of life. Our data suggest that the physiological, psychological and social changes occurring from childhood to adolescence might result in marked behavioral changes related to activity/sedentary levels. Most interesting is also that MVPA keep decreasing from adolescence to young adulthood, without changes in sedentary time during that period.

The decline in MVPA is in agreement with previous studies examining different age periods during childhood or adolescence: from 3 to 5 years (New Zealand)[25], from 5/6 to 7/8 years (Mexico)[29], from 7 to 9 years (UK)[26], from 9 to 15 years (USA)[31] and from 13 to 16 years (Vietnam)[28]. Only the study by Mitchell *et al*.[32] reported no change in MVPA in UK adolescents from the ALSPAC study followed from 12 to 16 years, but discrepancies can be due to the fact that the cut-point used to define MVPA (>3600 counts/min) was much higher than the one used in the rest of studies. Our study adds to the previous literature that the decline in MVPA observed from childhood to adolescence persists, yet less pronounced, from adolescence to young adulthood. Four previous studies reported an increase in sedentary time from 7 to 9 years (UK)[26], from 9 to 15 years (USA)[30], and from 12 to 16 years (UK [32] and Vietnam [44]). Our study contributes to these studies suggesting that the increase in sedentary time observed from childhood to adolescence might level-off after adolescence. It is plausible to think that part of the increase in sedentary time is due to more demanding school/academic requirements in adolescence and later compared with childhood, also to physiological changes leading to less spontaneous and intermittent PA. Our data suggest that promotion strategies should try to reduce the decline in MVPA at any age from childhood to young adulthood, while reduction in sedentary time should be particularly targeted in childhood, before it becomes a stable behavior.

### Changes in boys *vs.* girls

Both the decrease in MVPA and the increase in sedentary time were greater in boys than in girls, suggesting that the pattern of change in activity/sedentary time was “unhealthier” in boys compared with girls. This could partially explain previous findings from the EYHS in which we observed that boys were more likely to develop overweight than girls over a 6-year follow-up period, as measured by both BMI and body fat percentage[45]. In contrast, Nader *et al*.[31] observed similar changes in MVPA in US boys and girls followed from 9 to 15 years of age. Basterfield *et al*.[26] observed a decline in MVPA in UK girls but not in boys. Concerning sedentary time, studies in UK children[26] and adolescents[32] observed greater increase in sedentary time in girls than in boys, which is opposite to our results. Taking this findings together, we conclude that sex differences in changes in MVPA and sedentary time do not follow a consistent pattern and might be region-specific.

### Changes in MVPA *vs.* sedentary time

Our study provides a direct comparison on longitudinal changes in both MVPA and sedentary time and shows that the changes observed in sedentary time from childhood to adolescence are much larger in relative terms than those observed in MVPA. This finding has important public health implications, suggesting that prevention strategies focused at this period of life should consider as a major goal the reduction in sedentary time. Nevertheless, interventions focused on both reduction of sedentary time and increases in MVPA are desired and would obtain the greatest benefits on youth health. Most of studies focused only on one construct, i.e. MVPA[25], [28], [29], [31]. Three studies examined both MVPA and sedentary time[26], [30], [32]; however, since standardized z-scores were not used in their analyses it is complicated to do direct comparison of the magnitude of the change in both constructs.

### Changes in weekdays *vs.* weekend days

Children and adolescents are less active, yet also less sedentary, at weekends compared with weekdays. This pattern persists over the follow-up period of this study, with the largest differences taking place around adolescence. Similar trends were observed in US children followed from 9 to 15 years for MVPA[31]. It is plausible that during weekdays, children/adolescents are more sedentary due to sitting hours at the schools, homework, etc.; and they are also more active due to physical education sessions and extra-curricular organized PA.

### Limitations

Attrition and compliance might have biased our results. As in previous longitudinal studies on objective measured MVPA and/or sedentary time which included relatively long follow-up periods (4 to 6 years)[31], [32], participants with valid accelerometer data were more likely to be girls and to have mothers with high (university) educational level. The present study includes only two examinations, i.e. baseline and follow-up. Whenever is possible, the inclusion of more time points is desired.

### Clinical implications

The multiple benefits of an active lifestyle on health are well-known. Consequently, interventions focused on changes in activity/sedentary patterns in different population groups are on-going. The present study provides valuable information about changes in MVPA and sedentary time over important periods of life, which should assist researchers and health practitioners to better plan their interventions and set realistic goals.

### Conclusions

The results from this North-East European study cohort suggest that MVPA declines during the study period, more markedly from childhood to adolescence (overall change = 30 min/d) than from adolescence to young adulthood (overall change = 13 min/d). In addition, sedentary time largely increases from childhood to adolescence (overall change = 2∶45 h/d). The magnitude of the change is larger in boys compared with girls, and in sedentary time compared with MVPA. Activity and sedentary patterns differ between weekend days and weekdays, with the largest difference observed at adolescence. The decline in MVPA and increase in sedentary time observed are of concern and may increase the risk of developing obesity and other chronic diseases later in life. These findings substantially contribute to understand how key health-related behaviors (physical activity and sedentary) change across important periods of life.

## Supporting Information

Table S1 **Average number of valid days and registered time in each country, age-cohort and sex group, weekdays and weekend days separately.**
(DOC)

Table S2 **Mixed effect models examining the change in standardized (z-score) weekly (weekdays and weekend days weighted average) moderate-to-vigorous physical activity from childhood to adolescence and from adolescence to young adulthood in boys and girls.**
(DOC)

Table S3 **Mixed effect models examining the change in in standardized (z-score) weekly (weekdays and weekend days weighted average) sedentary time from childhood to adolescence and from adolescence to young adulthood in boys and girls.**
(DOC)

Table S4 **Mixed effect models examining the change in weekday moderate-to-vigorous physical activity from childhood to adolescence and from adolescence to young adulthood in boys and girls.**
(DOC)

Table S5 **Mixed effect models examining the change in weekend moderate-to-vigorous physical activity from childhood to adolescence and from adolescence to young adulthood in boys and girls.**
(DOC)

Table S6 **Mixed effect models examining the change in weekday sedentary time from childhood to adolescence and from adolescence to young adulthood in boys and girls.**
(DOC)

Table S7 **Mixed effect models examining the change in weekend sedentary time from childhood to adolescence and from adolescence to young adulthood in boys and girls.**
(DOC)

Methods S1 **Accelerometer methods and data analysis; Comparability between Actigraph accelerometers; Activity/inactivity cut-points used.**
(DOC)

References S1 **List of references cited in Methods S1.**
(DOC)
