# Supplementary material for: Objectively Measured Physical Activity and Sedentary Time during Childhood, Adolescence and Young Adulthood: A Cohort Study
Source: PLoS One. 2013 Apr 23;8(4):e60871. doi: 10.1371/journal.pone.0060871 (PMC3634054; doi:10.1371/journal.pone.0060871)
Supplement: Table S1 — Average number of valid days and registered time in each country, age-cohort and sex group, weekdays and weekend days separately. (DOC) [file pone.0060871.s001.doc]

**Table S1**. Average number of **valid days** and **registered time** in each country, age-cohort and sex group, **weekdays** and **weekend** days separately.

|  | **Swedish cohort** | | | | | | | | |  | **Estonian cohort** | | | | | | | | |
| --- | --- | --- | --- | --- | --- | --- | --- | --- | --- | --- | --- | --- | --- | --- | --- | --- | --- | --- | --- |
|  | **Young cohort** | | | | **Older cohort** | | | | |  | **Young cohort** | | | | | **Older cohort** | | | |
|  | **Boys** | | **Girls** | | **Boys** | | **Girls** | | |  | **Boys** | | | **Girls** | | **Boys** | | **Girls** | |
|  |  | |  | |  | |  | | |  |  | | |  | |  | |  | |
| Baseline/Follow-up data |  | |  | |  | |  | | |  |  | | |  | |  | |  | |
|  |  | |  | |  | |  | | |  |  | | |  | |  | |  | |
| **N** *  Baseline | 180 | | 213 | | 157 | | 203 | | |  | 214 | | | 220 | | 156 | | 223 | |
| Follow-up | 107 | | 133 | | 62 | | 106 | | |  | 114 | | | 149 | | 63 | | 90 | |
| **Weekdays** |  |  |  |  |  |  | |  |  |  |  |  |  | |  |  |  |  |  |
|  |  |  |  |  |  |  | |  |  |  |  |  |  | |  |  |  |  |  |
| **Valid days (no)** |  |  |  |  |  |  | |  |  |  |  |  |  | |  |  |  |  |  |
| Baseline | 2.1 | (0.3) | 2.0 | (0.1) | 2.1 | (0.2) | | 2.1 | (0.3) |  | 2.0 | (0.2) | 2.0 | | (0.2) | 2.0 | (0.2) | 2.0 | (0.2) |
| Follow-up † | 4.6 | (0.7) | 4.5 | (0.8) | 4.6 | (0.9) | | 4.6 | (0.8) |  | 2.1 | (0.4) | 2.1 | | (0.3) | 2.1 | (0.4) | 2.1 | (0.4) |
| **Registered time (h/d)** |  |  |  |  |  |  | |  |  |  |  |  |  | |  |  |  |  |  |
| Baseline | 13.8 | (1.3) | 13.8 | (1.0) | 14.7 | (1.2) | | 14.6 | (1.3) |  | 13.7 | (1.2) | 13.7 | | (1.2) | 14.2 | (1.5) | 14.4 | (1.3) |
| Follow-up | 14.1 | (1.8) | 14.2 | (2.0) | 14.5 | (2.2) | | 14.3 | (3.6) |  | 13.9 | (2.0) | 13.8 | | (1.8) | 13.7 | (1.7) | 13.2 | (1.8) |
|  |  |  |  |  |  |  | |  |  |  |  |  |  | |  |  |  |  |  |
| **Weekend days** |  |  |  |  |  |  | |  |  |  |  |  |  | |  |  |  |  |  |
|  |  |  |  |  |  |  | |  |  |  |  |  |  | |  |  |  |  |  |
| **Valid days (no)** |  |  |  |  |  |  | |  |  |  |  |  |  | |  |  |  |  |  |
| Baseline | 1.9 | (0.3) | 1.9 | (0.3) | 1.9 | (0.3) | | 1.9 | (0.3) |  | 1.9 | (0.2) | 1.9 | | (0.2) | 1.9 | (0.3) | 1.9 | (0.3) |
| Follow-up | 1.8 | (0.4) | 1.9 | (0.4) | 1.8 | (0.4) | | 1.8 | (0.4) |  | 1.8 | (0.4) | 1.8 | | (0.4) | 1.8 | (0.4) | 1.8 | (0.4) |
| **Registered time (h/d)** |  |  |  |  |  |  | |  |  |  |  |  |  | |  |  |  |  |  |
| Baseline | 12.7 | (1.5) | 12.6 | (1.8) | 13.2 | (1.8) | | 13.3 | (1.7) |  | 12.4 | (1.8) | 12.3 | | (1.3) | 12.7 | (1.9) | 12.5 | (1.8) |
| Follow-up | 12.6 | (2.4) | 13.0 | (3.4) | 13.4 | (3.4) | | 14.2 | (4.1) |  | 12.4 | (2.4) | 12.1 | | (1.9) | 12.7 | (2.2) | 12.5 | (1.9) |
|  |  |  |  |  |  |  | |  |  |  |  |  |  | |  |  |  |  |  |

Data are means (standard deviation), unless otherwise indicated. * The same sample size was available for all the variables shown. † Note that the number of valid days is larger in the follow-up because the Swedish participants in the follow-up examination were asked to wear the accelerometer for 7 days instead of 4 days (see Supporting information-Methods above).
