# Supplementary material for: Objectively Measured Physical Activity and Sedentary Time during Childhood, Adolescence and Young Adulthood: A Cohort Study
Source: PLoS One. 2013 Apr 23;8(4):e60871. doi: 10.1371/journal.pone.0060871 (PMC3634054; doi:10.1371/journal.pone.0060871)
Supplement: Table S2 — Mixed effect models examining the change in standardized (z-score) weekly (weekdays and weekend days weighted average) moderate-to-vigorous physical activity from childhood to adolescence and from adolescence to young adulthood in boys and girls. (DOC) [file pone.0060871.s002.doc]

**Table S2**. Mixed effect models examining the change in **standardized** (z-score) **weekly** (weekdays and weekend days weighted average) **moderate-to-vigorous** **physical** **activity** from childhood to adolescence and from adolescence to young adulthood in boys and girls.

|  |  |  | Boys |  |  |  |  | Girls |  |  |
| --- | --- | --- | --- | --- | --- | --- | --- | --- | --- | --- |
| Young cohort (N=960 ) |  | Coef. | 95% CI | | P |  | Coef. | 95% CI | | P |
|  |  |  |  |  |  |  |  |  |  |  |
| Intercept at baseline age (z-score) |  | -1.01 | -1.68 | -0.33 | 0.004 |  | -1.99 | -2.76 | -1.21 | <0.001 |
| Age (per year) † |  | -0.04 | -0.06 | -0.02 | <0.001 |  | -0.02 | -0.04 | 0.00 | 0.068 |
| Registered time (min/d) |  | 0.00 | 0.00 | 0.00 | <0.001 |  | 0.00 | 0.00 | 0.00 | <0.001 |
| Valid days (no.) |  | -0.04 | -0.13 | 0.06 | 0.456 |  | 0.01 | -0.11 | 0.12 | 0.930 |
| Country (Estonia=0, Sweden=1) |  | 0.41 | 0.20 | 0.62 | <0.001 |  | 0.41 | 0.21 | 0.60 | <0.001 |
| Age*country |  | -0.09 | -0.14 | -0.04 | 0.001 |  | -0.12 | -0.18 | -0.06 | <0.001 |
| Older cohort (N=840 ) |  | Coef. | 95% CI | | P |  | Coef. | 95% CI | | P |
|  |  |  |  |  |  |  |  |  |  |  |
| Intercept at baseline age (z-score) |  | -1.56 | -2.51 | -0.61 | 0.001 |  | -2.59 | -3.46 | -1.72 | <0.001 |
| Age (per year) † |  | -0.04 | -0.07 | -0.02 | <0.001 |  | -0.02 | -0.04 | 0.00 | 0.068 |
| Registered time (min/d) |  | 0.00 | 0.00 | 0.00 | <0.001 |  | 0.00 | 0.00 | 0.00 | <0.001 |
| Valid days (no.) |  | -0.18 | -0.32 | -0.04 | 0.010 |  | -0.09 | -0.23 | 0.04 | 0.170 |
| Country (Estonia=0, Sweden=1) |  | 0.07 | -0.16 | 0.29 | 0.569 |  | 0.34 | 0.15 | 0.52 | <0.001 |
| Age*country |  | 0.04 | -0.03 | 0.11 | 0.256 |  | -0.01 | -0.08 | 0.06 | 0.802 |

† Age was centered on age at baseline. The coefficient (confidence intervals, CI) is interpreted as change in physical activity (standard deviations) per year of follow-up. Mean (min-max) follow-up period was 7.5 (4.9-9.4) years and 7.9 (5.7-10.3) in the young cohort and older cohort respectively.
