# Supplementary material for: Objectively Measured Physical Activity and Sedentary Time during Childhood, Adolescence and Young Adulthood: A Cohort Study
Source: PLoS One. 2013 Apr 23;8(4):e60871. doi: 10.1371/journal.pone.0060871 (PMC3634054; doi:10.1371/journal.pone.0060871)
Supplement: Table S3 — Mixed effect models examining the change in in standardized (z-score) weekly (weekdays and weekend days weighted average) sedentary time from childhood to adolescence and from adolescence to young adulthood in boys and girls. (DOC) [file pone.0060871.s003.doc]

**Table S3**. Mixed effect models examining the change in in **standardized** (z-score) **weekly** (weekdays and weekend days weighted average) **sedentary** **time** from childhood to adolescence and from adolescence to young adulthood in boys and girls.

|  |  |  | Boys |  |  |  |  | Girls |  |  |
| --- | --- | --- | --- | --- | --- | --- | --- | --- | --- | --- |
| Young cohort (N=960 ) |  | Coef. | 95% CI | | P |  | Coef. | 95% CI | | P |
|  |  |  |  |  |  |  |  |  |  |  |
| Intercept at baseline age (z-score) |  | -3.54 | -4.07 | -3.01 | <0.001 |  | -3.49 | -3.98 | -3.00 | <0.001 |
| Age (per year) † |  | 0.14 | 0.12 | 0.15 | <0.001 |  | 0.12 | 0.10 | 0.13 | <0.001 |
| Registered time (min/d) |  | 0.00 | 0.00 | 0.00 | <0.001 |  | 0.00 | 0.00 | 0.00 | <0.001 |
| Valid days (no.) |  | 0.02 | -0.05 | 0.09 | 0.542 |  | 0.04 | -0.03 | 0.11 | 0.282 |
| Country (Estonia=0, Sweden=1) |  | -0.11 | -0.28 | 0.06 | 0.201 |  | -0.41 | -0.58 | -0.24 | <0.001 |
| Age*country ‡ |  | 0.05 | 0.01 | 0.09 | 0.017 |  | 0.09 | 0.05 | 0.13 | <0.001 |
| Older cohort (N=840 ) |  | Coef. | 95% CI | | P |  | Coef. | 95% CI | | P |
|  |  |  |  |  |  |  |  |  |  |  |
| Intercept at baseline age (z-score) |  | -3.37 | -4.39 | -2.35 | <0.001 |  | -3.88 | -4.82 | -2.93 | <0.001 |
| Age (per year) † |  | 0.03 | 0.00 | 0.05 | 0.038 |  | 0.01 | -0.01 | 0.03 | 0.281 |
| Registered time (min/d) |  | 0.00 | 0.00 | 0.00 | <0.001 |  | 0.00 | 0.00 | 0.01 | <0.001 |
| Valid days (no.) |  | 0.08 | -0.06 | 0.23 | 0.269 |  | 0.14 | -0.01 | 0.29 | 0.076 |
| Country (Estonia=0, Sweden=1) |  | 0.12 | -0.10 | 0.35 | 0.282 |  | -0.21 | -0.40 | -0.02 | 0.031 |
| Age*country |  | -0.01 | -0.08 | 0.07 | 0.873 |  | -0.02 | -0.10 | 0.05 | 0.582 |

† Age was centered on age at baseline. The coefficient (confidence intervals, CI) is interpreted as change in sedentary time (standard deviations) per year of follow-up. Mean (min-max) follow-up period was 7.5 (4.9-9.4) years and 7.9 (5.7-10.3) in the young cohort and older cohort respectively.
