# Supplementary material for: Objectively Measured Physical Activity and Sedentary Time during Childhood, Adolescence and Young Adulthood: A Cohort Study
Source: PLoS One. 2013 Apr 23;8(4):e60871. doi: 10.1371/journal.pone.0060871 (PMC3634054; doi:10.1371/journal.pone.0060871)
Supplement: Table S4 — Mixed effect models examining the change in weekday moderate-to-vigorous physical activity from childhood to adolescence and from adolescence to young adulthood in boys and girls. (DOC) [file pone.0060871.s004.doc]

**Table S4**. Mixed effect models examining the change in **weekday** **moderate-to-vigorous** **physical** **activity** from childhood to adolescence and from adolescence to young adulthood in boys and girls.

|  |  |  | Boys |  |  |  |  | Girls |  |  |
| --- | --- | --- | --- | --- | --- | --- | --- | --- | --- | --- |
| Young cohort (N=960 ) |  | Coef. | 95% CI | | P |  | Coef. | 95% CI | | P |
|  |  |  |  |  |  |  |  |  |  |  |
| Intercept at baseline age (min/d) |  | 34.1 | 2.8 | 65.4 | 0.033 |  | -3.4 | -28.8 | 21.9 | 0.790 |
| Age (per year) † |  | -1.9 | -3.0 | -0.8 | 0.001 |  | -0.6 | -1.5 | 0.2 | 0.143 |
| Registered time (min/d) |  | 0.1 | 0.0 | 0.1 | <0.001 |  | 0.1 | 0.1 | 0.1 | <0.001 |
| Valid days (no.) |  | -3.2 | -9.3 | 2.9 | 0.300 |  | -1.0 | -6.0 | 4.1 | 0.701 |
| Country (Estonia=0, Sweden=1) |  | 22.5 | 11.9 | 33.1 | <0.001 |  | 14.1 | 6.7 | 21.5 | <0.001 |
| Age*country ‡ |  | -4.6 | -7.7 | -1.5 | 0.004 |  | -4.0 | -6.5 | -1.5 | 0.002 |
| Older cohort (N=840 ) |  | Coef. | 95% CI | | P |  | Coef. | 95% CI | | P |
|  |  |  |  |  |  |  |  |  |  |  |
| Intercept at baseline age (min/d) |  | -5.7 | -43.9 | 32.5 | 0.770 |  | -41.3 | -65.6 | -16.9 | 0.001 |
| Age (per year) † |  | -2.3 | -3.4 | -1.2 | <0.001 |  | -0.8 | -1.6 | 0.0 | 0.056 |
| Registered time (min/d) |  | 0.1 | 0.1 | 0.2 | <0.001 |  | 0.1 | 0.1 | 0.1 | <0.001 |
| Valid days (no.) |  | -9.3 | -17.1 | -1.5 | 0.019 |  | 0.0 | -4.7 | 4.6 | 0.997 |
| Country (Estonia=0, Sweden=1) |  | -1.8 | -11.7 | 8.1 | 0.721 |  | 8.7 | 2.5 | 14.9 | 0.006 |
| Age*country ‡ |  | 3.1 | -0.7 | 6.8 | 0.110 |  | -1.8 | -4.0 | 0.5 | 0.126 |

† Age was centered on age at baseline. The coefficient (confidence intervals, CI) is interpreted as change in physical activity (min/d) per year of follow-up. Mean (min-max) follow-up period was 7.5 (4.9-9.4) years and 7.9 (5.7-10.3) in the young cohort and older cohort respectively.

‡ The coefficient for age*country interaction term is interpreted as follows: e.g. Coef= -4.6, physical activity decreased 4.6 min/d more in Swedish participants compared with Estonian participants per year of follow-up.
