# Supplementary material for: Objectively Measured Physical Activity and Sedentary Time during Childhood, Adolescence and Young Adulthood: A Cohort Study
Source: PLoS One. 2013 Apr 23;8(4):e60871. doi: 10.1371/journal.pone.0060871 (PMC3634054; doi:10.1371/journal.pone.0060871)
Supplement: Table S5 — Mixed effect models examining the change in weekend moderate-to-vigorous physical activity from childhood to adolescence and from adolescence to young adulthood in boys and girls. (DOC) [file pone.0060871.s005.doc]

**Table S5**. Mixed effect models examining the change in **weekend** **moderate-to-vigorous** **physical** **activity** from childhood to adolescence and from adolescence to young adulthood in boys and girls.

|  |  |  | Boys |  |  |  |  | Girls |  |  |
| --- | --- | --- | --- | --- | --- | --- | --- | --- | --- | --- |
| Young cohort (N=960 ) |  | Coef. | 95% CI | | P |  | Coef. | 95% CI | | P |
|  |  |  |  |  |  |  |  |  |  |  |
| Intercept at baseline age (min/d) |  | 9.7 | -19.0 | 38.3 | 0.508 |  | 4.2 | -17.0 | 25.4 | 0.700 |
| Age (per year) † |  | -3.8 | -5.0 | -2.7 | <0.001 |  | -2.1 | -3.0 | -1.2 | <0.001 |
| Registered time (min/d) |  | 0.1 | 0.1 | 0.1 | <0.001 |  | 0.1 | 0.1 | 0.1 | <0.001 |
| Valid days (no.) |  | -0.5 | -10.2 | 9.1 | 0.913 |  | -2.6 | -10.3 | 5.2 | 0.518 |
| Country (Estonia=0, Sweden=1) |  | 11.3 | -0.2 | 22.7 | 0.054 |  | 4.8 | -3.2 | 12.7 | 0.239 |
| Age*country ‡ |  | -3.0 | -5.0 | -1.0 | 0.003 |  | -1.9 | -3.3 | -0.5 | 0.007 |
| Older cohort (N=840 ) |  | Coef. | 95% CI | | P |  | Coef. | 95% CI | | P |
|  |  |  |  |  |  |  |  |  |  |  |
| Intercept at baseline age (min/d) |  | 11.8 | -17.3 | 41.0 | 0.427 |  | -3.9 | -24.2 | 16.3 | 0.702 |
| Age (per year) † |  | -1.7 | -2.9 | -0.5 | 0.004 |  | -0.9 | -1.6 | -0.2 | 0.007 |
| Registered time (min/d) |  | 0.1 | 0.0 | 0.1 | <0.001 |  | 0.1 | 0.0 | 0.1 | <0.001 |
| Valid days (no.) |  | -4.4 | -14.6 | 5.9 | 0.403 |  | -2.2 | -9.2 | 4.8 | 0.542 |
| Country (Estonia=0, Sweden=1) |  | 1.7 | -8.8 | 12.1 | 0.753 |  | 6.3 | 0.2 | 12.5 | 0.043 |
| Age*country ‡ |  | -1.7 | -3.7 | 0.3 | 0.101 |  | -0.1 | -1.4 | 1.2 | 0.871 |

† Age was centered on age at baseline. The coefficient (confidence intervals, CI) is interpreted as change in physical activity (min/d) per year of follow-up. Mean (min-max) follow-up period was 7.5 (4.9-9.4) years and 7.9 (5.7-10.3) in the young cohort and older cohort respectively.

‡ The coefficient for age*country interaction term is interpreted as follows: e.g. Coef= -3.0, physical activity decreased 3 min/d more in Swedish participants compared with Estonian participants per year of follow-up.
