# Supplementary material for: Objectively Measured Physical Activity and Sedentary Time during Childhood, Adolescence and Young Adulthood: A Cohort Study
Source: PLoS One. 2013 Apr 23;8(4):e60871. doi: 10.1371/journal.pone.0060871 (PMC3634054; doi:10.1371/journal.pone.0060871)
Supplement: Table S6 — Mixed effect models examining the change in weekday sedentary time from childhood to adolescence and from adolescence to young adulthood in boys and girls. (DOC) [file pone.0060871.s006.doc]

**Table S6**. Mixed effect models examining the change in **weekday** **sedentary** **time** from childhood to adolescence and from adolescence to young adulthood in boys and girls.

|  |  |  | Boys |  |  |  |  | Girls |  |  |
| --- | --- | --- | --- | --- | --- | --- | --- | --- | --- | --- |
| Young cohort (N=960 ) |  | Coef. | 95% CI | | P |  | Coef. | 95% CI | | P |
|  |  |  |  |  |  |  |  |  |  |  |
| Intercept at baseline age (min/d) |  | -179.6 | -254.9 | -104.2 | <0.001 |  | -66.3 | -129.0 | -3.6 | 0.038 |
| Age (per year) † |  | 20.1 | 17.5 | 22.6 | <0.001 |  | 15.5 | 13.4 | 17.7 | <0.001 |
| Registered time (min/d) |  | 0.6 | 0.5 | 0.7 | <0.001 |  | 0.5 | 0.5 | 0.6 | <0.001 |
| Valid days (no.) |  | 9.0 | -5.7 | 23.8 | 0.230 |  | -0.2 | -12.5 | 12.2 | 0.980 |
| Country (Estonia=0, Sweden=1) |  | -32.9 | -56.3 | -9.5 | 0.006 |  | -72.6 | -94.0 | -51.2 | <0.001 |
| Age*country ‡ |  | 6.7 | -0.6 | 14.1 | 0.072 |  | 15.8 | 9.5 | 22.0 | <0.001 |
| Older cohort (N=840 ) |  | Coef. | 95% CI | | P |  | Coef. | 95% CI | | P |
|  |  |  |  |  |  |  |  |  |  |  |
| Intercept at baseline age (min/d) |  | 133.7 | 21.4 | 245.9 | 0.020 |  | 156.3 | 71.8 | 240.8 | <0.001 |
| Age (per year) † |  | 2.5 | -0.7 | 5.8 | 0.124 |  | 0.2 | -2.0 | 2.5 | 0.835 |
| Registered time (min/d) |  | 0.4 | 0.3 | 0.5 | <0.001 |  | 0.4 | 0.3 | 0.5 | <0.001 |
| Valid days (no.) |  | -14.2 | -37.4 | 9.0 | 0.229 |  | -1.3 | -19.2 | 16.6 | 0.884 |
| Country (Estonia=0, Sweden=1) |  | 6.8 | -20.6 | 34.3 | 0.625 |  | -31.9 | -51.7 | -12.0 | 0.002 |
| Age*country ‡ |  | 10.1 | -0.9 | 21.1 | 0.072 |  | 4.5 | -3.8 | 12.9 | 0.289 |

† Age was centered on age at baseline. The coefficient (confidence intervals, CI) is interpreted as change in sedentary time (min/d) per year of follow-up. Mean (min-max) follow-up period was 7.5 (4.9-9.4) years and 7.9 (5.7-10.3) in the young cohort and older cohort respectively.

‡ The coefficient for age*country interaction term is interpreted as follows: e.g. Coef=6.7, sedentary time increased 6.7 min/d more in Swedish participants compared with Estonian participants per year of follow-up.
