# Supplementary material for: Objectively Measured Physical Activity and Sedentary Time during Childhood, Adolescence and Young Adulthood: A Cohort Study
Source: PLoS One. 2013 Apr 23;8(4):e60871. doi: 10.1371/journal.pone.0060871 (PMC3634054; doi:10.1371/journal.pone.0060871)
Supplement: Table S7 — Mixed effect models examining the change in weekend sedentary time from childhood to adolescence and from adolescence to young adulthood in boys and girls. (DOC) [file pone.0060871.s007.doc]

**Table S7**. Mixed effect models examining the change in **weekend** **sedentary** **time** from childhood to adolescence and from adolescence to young adulthood in boys and girls.

|  |  |  | Boys |  |  |  |  | Girls |  |  |
| --- | --- | --- | --- | --- | --- | --- | --- | --- | --- | --- |
| Young cohort (N=960 ) |  | Coef. | 95% CI | | P |  | Coef. | 95% CI | | P |
|  |  |  |  |  |  |  |  |  |  |  |
| Intercept at baseline age (min/d) |  | -99.6 | -166.9 | -32.4 | 0.004 |  | -94.6 | -150.0 | -39.1 | 0.001 |
| Age (per year) † |  | 19.5 | 16.9 | 22.1 | <0.001 |  | 15.0 | 12.9 | 17.1 | <0.001 |
| Registered time (min/d) |  | 0.5 | 0.4 | 0.6 | <0.001 |  | 0.5 | 0.4 | 0.5 | <0.001 |
| Valid days (no.) |  | -0.2 | -22.7 | 22.3 | 0.988 |  | 25.6 | 5.5 | 45.6 | 0.012 |
| Country (Estonia=0, Sweden=1) |  | 0.1 | -24.6 | 24.7 | 0.996 |  | -26.2 | -47.9 | -4.6 | 0.018 |
| Age*country ‡ |  | 9.1 | 4.7 | 13.5 | <0.001 |  | 10.7 | 7.2 | 14.2 | <0.001 |
| Older cohort (N=840 ) |  | Coef. | 95% CI | | P |  | Coef. | 95% CI | | P |
|  |  |  |  |  |  |  |  |  |  |  |
| Intercept at baseline age (min/d) |  | -16.7 | -101.8 | 68.3 | 0.700 |  | 23.0 | -41.3 | 87.4 | 0.483 |
| Age (per year) † |  | 4.1 | 0.8 | 7.4 | 0.014 |  | 2.8 | 0.7 | 5.0 | 0.009 |
| Registered time (min/d) |  | 0.4 | 0.4 | 0.5 | <0.001 |  | 0.5 | 0.4 | 0.6 | <0.001 |
| Valid days (no.) |  | 36.9 | 7.7 | 66.2 | 0.013 |  | 13.4 | -8.9 | 35.6 | 0.238 |
| Country (Estonia=0, Sweden=1) |  | 24.8 | -2.4 | 52.1 | 0.074 |  | -4.1 | -23.8 | 15.5 | 0.681 |
| Age*country ‡ |  | 1.5 | -3.9 | 6.8 | 0.588 |  | 3.6 | -0.4 | 7.7 | 0.077 |

† Age was centered on age at baseline. The coefficient (confidence intervals, CI) is interpreted as change in sedentary time (min/d) per year of follow-up. Mean (min-max) follow-up period was 7.5 (4.9-9.4) years and 7.9 (5.7-10.3) in the young cohort and older cohort respectively.

‡ The coefficient for age*country interaction term is interpreted as follows: e.g. Coef=9.1, sedentary time increased 9.1 min/d more in Swedish participants compared with Estonian participants per year of follow-up.
