# Supplementary material for: Objectively Measured Physical Activity and Sedentary Time during Childhood, Adolescence and Young Adulthood: A Cohort Study
Source: PLoS One. 2013 Apr 23;8(4):e60871. doi: 10.1371/journal.pone.0060871 (PMC3634054; doi:10.1371/journal.pone.0060871)
Supplement: Methods S1 — Accelerometer methods and data analysis; Comparability between Actigraph accelerometers; Activity/inactivity cut-points used. (DOC) [file pone.0060871.s008.doc]

**SUPPORTING INFORMATION-METHODS**

**Accelerometer methods and data analysis**

At baseline, Estonian and Swedish participants were instructed to wear the monitor continuously during waking hours (except when doing water-based activities), for 4 consecutive days, including 2 weekdays and 2 weekend days. In Sweden, participants at follow-up were asked to wear the accelerometers for 7 consecutive days. Although reliability of activity measures do not differ much between 4 *vs*. 6-7 days in youth (Intra-class correlation coefficient=0.40 *vs*. 0.46, respectively), all the analyses were additionally adjusted for number of valid days to account for a potential confounding effect due to compliance, i.e. fewer days of valid recording have shown to be related with modestly higher activity levels. Inclusion criteria were: to have at least 2 valid weekdays and 1 valid weekend day, defining a valid day as 10h or more of registered time. High compliance was observed in this study; i.e. 93.2% of the participants had 4 valid days or more at baseline, 82.5% of the Estonian participants had 4 or more valid days at follow-up and 83.3% of the Swedish participants had 6/7 valid days at follow-up. **Table S1** shows the average number of valid days and registered time in each country, age-cohort and sex group, weekdays and weekend days separately.

As standard when using the AM7164 accelerometer (due to the limited storage capacity of this early generation accelerometer, the activity counts detected by the accelerometer were averaged and stored every 60 seconds (epoch). Because children typically perform PA intermittently in short bursts lasting several seconds, when using 60 seconds epoch a small underestimation on MVPA levels need to be assumed.

In the present study, 20min of consecutive zeros were considered as non-wearing time and were removed from the analyses. This criteria lays within the values most frequently used in the literature in children (10 min) and in adults (60 min), which is a good compromise for the current study that follows individuals during childhood, adolescence and young adulthood.

**Comparability between Actigraph accelerometers: AM7164 *vs*. GT1M**

Activity monitors are in continuous development and different accelerometers will often be used at baseline and follow-up in longitudinal studies. Comparability between accelerometer models is an important issue that could potentially account for some of the changes observed in PA and sedentary time. In this context, Corder *et al*. examined the agreement in free-living PA and sedentary time between AM7164 and GT1M accelerometers in youth. They observed a good agreement between these two accelerometer models for MVPA, suggesting that our findings on MVPA should not be affected by the use of two different accelerometer models at baseline and follow-up. The authors also reported that GT1M measured an average of 24 min/d more in sedentary time than model 7164 did, suggesting that the changes in sedentary time reported in our study could be potentially overestimated in 24min. However, it is important to note that in Corder *et al*.’ study , non-wearing periods and sleeping time were included as sedentary time. If non-wearing periods and sleeping time would have been excluded from the analyses, as done in our study and most of studies, total sedentary time would have been smaller and the inter-monitor differences consequently also smaller than the reported 24min/d. Nevertheless, the size of the change (follow-up – baseline) observed in sedentary time in our study was large, 2 to 3:30h, so that the conclusions of the present study remain solid, even in the hypothetical case of a 24min/d inter-monitor variability.

**Activity/inactivity cut-points used: rationale**

The selection of one or other cut-points is an important issue in studies using accelerometry. The literature is rather consistent about the cut-point for sedentary time, i.e. ≤100 counts/min, in children, adolescents and adults; except for the ALSPAC study, which used 200 counts/min as cut-point for sedentary activities, because the software they used to analyze accelerometer data did not allow setting lower thresholds. We used the cut-point of 100 counts/min to be consistent with most of literature and to enhance future comparability.

A more complex issue is however the selection of cut-points for PA at different intensities, which have been validated in either children or adults. Since this is a longitudinal study following individuals from young ages to early adulthood, the selection of the most appropriate cut-point become more complicated. The use of different cut-points at baseline and follow-up might not be a good decision, since changes observed in PA could be due to differences in the intensity thresholds rather than actual changes in PA. As an example, Nader *et al*. used Freedson's cut-points for children/adolescents . A limitation of Freedson's cut-points when applied to longitudinal studies in youth is that they are age-dependent. Consequently, the absolute cut-point for moderate-to-vigorous PA (MVPA) used in the study by Nader *et al*. at baseline (9 year-olds) and follow-up (15 year-olds) is markedly different (910 *vs*. 1710 counts per minute, respectively) and is unknown to what extent the higher cut-point used at follow-up explains the decrease observed from baseline to follow-up in MVPA. Of note is also that Nader *et al*., considered moderate PA when the estimated energy expenditure of the activity was equal or higher than 3 METs (metabolic equivalents); however, the 3 METs threshold is more commonly used in adult and there is consistent evidence that brisk walking, a key behavioral indicator of moderate intensity PA, is associated with an energy cost of approximately 4 METs in children and adolescents .

Regarding cut-points in children, Trost *et al*. have recently tested the 5 most commonly used cut-points available in the literature and oncluded that, for children and adolescents, the best prediction, as well as specificity and sensitivity, of time spent at different intensities was provided by Evenson's cut-points. Even if these cut-points were originally validated in a group of young individuals (5 to 9 year-olds), the validation study conducted by Trost *et al*. observed that they had the best performance in all age groups (range from 5 to 15 year-olds). For adults, the Freedson's cut-points have been consistently used in the literature . In addition, they are especially appropriate for the purposes of the present study, since they were originally validated in young adults (23 year-olds), which perfectly fit with the age of our older cohort at follow-up.

Taking these into account, our decision was to use the cut-point of ≥2000 counts/min to define MVPA that falls within the cut-point recommended for children/adolescents (i.e. Evenson’s cut-point=2296 counts/min) and that recommended for adults (Freedson’s cut-point=1952 counts/min). The cut-point of 2000 counts/min for MVPA has been largely used in similar epidemiological studies conducted on young people.
