# Supplementary material for: Objectively Measured Physical Activity and Sedentary Time during Childhood, Adolescence and Young Adulthood: A Cohort Study
Source: PLoS One. 2013 Apr 23;8(4):e60871. doi: 10.1371/journal.pone.0060871 (PMC3634054; doi:10.1371/journal.pone.0060871)
Supplement: References S1 — List of references cited in Methods S1. (DOC) [file pone.0060871.s009.doc]

**SUPPORTING INFORMATION-REFERENCES**

1. Mattocks C, Leary S, Ness A, Deere K, Saunders J, et al. (2007) Intraindividual variation of objectively measured physical activity in children. Med Sci Sports Exerc 39: 622-629.

2. Mattocks C, Ness A, Leary S, Tilling K, Blair SN, et al. (2008) Use of accelerometers in a large field-based study of children: protocols, design issues, and effects on precision. J Phys Act Health 5 Suppl 1: S98-111.

3. Ruiz JR, Rizzo NS, Hurtig-Wennlöf A, Ortega FB, Warnberg J, et al. (2006) Relations of total physical activity and intensity to fitness and fatness in children; The European Youth Heart Study. Am J Clin Nutr 84: 298-302.

4. Ruiz JR, Ortega FB, Martinez-Gomez D, Labayen I, Moreno LA, et al. (2011) Objectively Measured Physical Activity and Sedentary Time in European Adolescents: The HELENA Study. Am J Epidemiol.

5. Riddoch CJ, Bo Andersen L, Wedderkopp N, Harro M, Klasson-Heggebo L, et al. (2004) Physical activity levels and patterns of 9- and 15-yr-old European children. Med Sci Sports Exerc 36: 86-92.

6. Matthews CE, Chen KY, Freedson PS, Buchowski MS, Beech BM, et al. (2008) Amount of time spent in sedentary behaviors in the United States, 2003-2004. Am J Epidemiol 167: 875-881.

7. Riddoch CJ, Mattocks C, Deere K, Saunders J, Kirkby J, et al. (2007) Objective measurement of levels and patterns of physical activity. Arch Dis Child.

8. Hagstromer M, Oja P, Sjostrom M (2007) Physical activity and inactivity in an adult population assessed by accelerometry. Med Sci Sports Exerc 39: 1502-1508.

9. Troiano RP, Berrigan D, Dodd KW, Masse LC, Tilert T, et al. (2008) Physical activity in the United States measured by accelerometer. Med Sci Sports Exerc 40: 181-188.

10. Mitchell JA, Pate RR, Dowda M, Mattocks C, Riddoch C, et al. (2011) A Prospective Study of Sedentary Behavior in a Large Cohort of Youth. Med Sci Sports Exerc.

11. Trost SG, McIver KL, Pate RR (2005) Conducting accelerometer-based activity assessments in field-based research. Med Sci Sports Exerc 37: S531-543.

12. Riddoch CJ, Leary SD, Ness AR, Blair SN, Deere K, et al. (2009) Prospective associations between objective measures of physical activity and fat mass in 12-14 year old children: the Avon Longitudinal Study of Parents and Children (ALSPAC). BMJ 339: b4544.

13. Hagstromer M, Troiano RP, Sjostrom M, Berrigan D (2010) Levels and patterns of objectively assessed physical activity--a comparison between Sweden and the United States. Am J Epidemiol 171: 1055-1064.

14. Corder K, Brage S, Ramachandran A, Snehalatha C, Wareham N, et al. (2007) Comparison of two Actigraph models for assessing free-living physical activity in Indian adolescents. J Sports Sci 25: 1607-1611.

15. Trost SG, Loprinzi PD, Moore R, Pfeiffer KA (2011) Comparison of accelerometer cut points for predicting activity intensity in youth. Med Sci Sports Exerc 43: 1360-1368.

16. Nader PR, Bradley RH, Houts RM, McRitchie SL, O'Brien M (2008) Moderate-to-vigorous physical activity from ages 9 to 15 years. JAMA 300: 295-305.

17. Freedson P, Pober D, Janz KF (2005) Calibration of accelerometer output for children. Med Sci Sports Exerc 37: S523-530.

18. Mattocks C, Leary S, Ness A, Deere K, Saunders J, et al. (2007) Calibration of an accelerometer during free-living activities in children. Int J Pediatr Obes 2: 218-226.

19. Ridley K, Ainsworth BE, Olds TS (2008) Development of a compendium of energy expenditures for youth. Int J Behav Nutr Phys Act 5: 45.

20. Treuth MS, Schmitz K, Catellier DJ, McMurray RG, Murray DM, et al. (2004) Defining accelerometer thresholds for activity intensities in adolescent girls. Med Sci Sports Exerc 36: 1259-1266.

21. Pate RR, Stevens J, Pratt C, Sallis JF, Schmitz KH, et al. (2006) Objectively measured physical activity in sixth-grade girls. Arch Pediatr Adolesc Med 160: 1262-1268.

22. Evenson KR, Catellier DJ, Gill K, Ondrak KS, McMurray RG (2008) Calibration of two objective measures of physical activity for children. J Sports Sci 26: 1557-1565.

23. Freedson PS, Melanson E, Sirard J (1998) Calibration of the Computer Science and Applications, Inc. accelerometer. Med Sci Sports Exerc 30: 777-781.

24. Andersen LB, Harro M, Sardinha LB, Froberg K, Ekelund U, et al. (2006) Physical activity and clustered cardiovascular risk in children: a cross-sectional study (The European Youth Heart Study). Lancet 368: 299-304.

25. Steele RM, van Sluijs EM, Cassidy A, Griffin SJ, Ekelund U (2009) Targeting sedentary time or moderate- and vigorous-intensity activity: independent relations with adiposity in a population-based sample of 10-y-old British children. Am J Clin Nutr 90: 1185-1192.

26. Ekelund U, Sardinha LB, Anderssen SA, Harro M, Franks PW, et al. (2004) Associations between objectively assessed physical activity and indicators of body fatness in 9- to 10-y-old European children: a population-based study from 4 distinct regions in Europe (the European Youth Heart Study). Am J Clin Nutr 80: 584-590.
